# Optical Feedback Interferometry for Velocity Measurement of Parallel Liquid-Liquid Flows in a Microchannel

**DOI:** 10.3390/s16081233

**Published:** 2016-08-04

**Authors:** Evelio E. Ramírez-Miquet, Julien Perchoux, Karine Loubière, Clément Tronche, Laurent Prat, Oscar Sotolongo-Costa

**Affiliations:** 1 LAAS-CNRS, Université de Toulouse, CNRS, INP, Toulouse F-31400, France; eramirez@inp-toulouse.fr (E.E.R.-M.); clement.tronche@enseeiht.fr (C.T.); 2 Centro de Aplicaciones Tecnológicas y Desarrollo Nuclear (CEADEN), Calle 30 No. 502, Miramar, La Habana 11300, Cuba; 3 Laboratoire de Génie Chimique, Université de Toulouse, CNRS, INPT, UPS, 4 allée Emile Monso, BP 84234, Toulouse F-31432, France; karine.loubiere@ensiacet.fr (K.L.); directeur@ensiacet.fr (L.P.); 4 Instituto de Investigación en Ciencias Básicas y Aplicadas, Universidad Autónoma del Estado de Morelos, Cuernavaca 62209, Mexico; osotolongo@uaem.mx; 5 Cátedra de Sistemas Complejos Henri Poincaré, Universidad de La Habana, La Habana 10400, Cuba

**Keywords:** optical feedback interferometry, velocimetry, microfluidics, parallel two-phase flows

## Abstract

Optical feedback interferometry (OFI) is a compact sensing technique with recent implementation for flow measurements in microchannels. We propose implementing OFI for the analysis at the microscale of multiphase flows starting with the case of parallel flows of two immiscible fluids. The velocity profiles in each phase were measured and the interface location estimated for several operating conditions. To the authors knowledge, this sensing technique is applied here for the first time to multiphase flows. Theoretical profiles issued from a model based on the Couette viscous flow approximation reproduce fairly well the experimental results. The sensing system and the analysis presented here provide a new tool for studying more complex interactions between immiscible fluids (such as liquid droplets flowing in a microchannel).

## 1. Introduction

Microfluidic systems offer a complete platform to control and observe chemical and biomedical processes that are too complex to be studied at the macroscale. Typical microscale devices allow for flow assessment in the laminar regime, where experimentation and processes can be easily controlled. Therefore, the microfluidic devices and the lab-on-chip systems open up new perspectives for applications in applied physics, chemistry and biology. In microstructured technologies, multiple-phase processes such as emulsification, polymerization, extraction, absorption or multiphase chemical reactions, benefit from the enhanced transport phenomena, the low substance consumption and reduced and highly controlled experimentation [1].

The configuration where two immiscible substances are flowing in the same microchannel occurs in many chemical and biochemical systems [2,3,4,5]. In this regard, the formation of droplets in microfluidic devices and the hydrodynamics of slug flows have received particular attention [6]. The gold standard to characterize the interaction of two liquid flows at the microscale is the analysis of flow patterns, but there remains a large variety of liquid–liquid interactions to be properly explored [7].

To understand the liquid-liquid interactions (two-phase flow structure, mixing, mass transfer, etc.), hydrodynamics parameters have to be determined. However, measuring velocities in small dimension channels with acceptable accuracy is challenging. At present, the conventional technique to measure velocity fields at microscale is the Micro Particle Image Velocimetry (µ-PIV). However, currently available µ-PIV systems in their minimal configuration [8] include a bulky high-power pulsed Nd:YAG laser, a fast acquisition camera and a microscope system. The tracers in the fluid have to be fluorescent particles. The optical arrangement in µ-PIV requires that the vision field of the camera and the laser focus be in perfect correspondence and then implies a robust and precise alignment of all the opto-mechanical assembly. Thus, µ-PIV are then heavy and expensive systems.

Optical feedback interferometry (OFI) has proven to be a strong alternative to the µ-PIV sensing method for velocity measurements [9]. In addition, it has demonstrated offering a good agreement with the “dual-slit” imaging technique [10] for the reconstruction of flow profiles in microchannels [11]. OFI uses an extremely compact interferometric scheme where a laser diode is used as the emitter, the interferometer and the receiver. In addition, OFI sensors require a minimal optical component arrangement and are by nature self-aligned, as it is not required to align two different optical systems. OFI sensors can measure local velocity with similar spatial resolution than usual interferometric techniques, thus being a suitable alternative for sensing at the microscale. Despite the fact that it is not intrinsically an imaging technique, the usual laser scanning system can be deployed to provide Doppler images of flows.

As a first approach to immiscible fluids interactions, we focused our attention on velocity measurements of oil-water parallel flows in a Y-shaped microreactor, as such flows are the simplest case of liquid-liquid interactions. In this work, the potentiality of the OFI sensing technique is tested to characterize the flows and to estimate the location of the interface separating both fluids in the microchannel. We present experimental results of velocity profile measurements and explore the impact of changes in the water flow rate, the latter providing valuable quantitative information on the spatial repartition of the fluids. In addition, we use the OFI sensing scheme to interrogate the flow profiles while keeping constant the ratio of flow rates imposed at the inlets. Under such conditions, the interface position is expected to remain unchanged. The interaction of both immiscible fluids is mainly influenced by the pressure gradient and viscosity in the kinetics of each parallel flow. As a consequence, a theoretical model that considers oil and water as viscous fluids is proposed to describe the the possible influence of one phase on the other. It is based on the Couette flows approximation and is compared to the experimental results.

The work presented here shows that the OFI sensing method allows for accurately measuring local velocity of two-phase parallel flows and studying flow interactions at the microscale.

## 2. Optical Feedback Interferometry

Optical feedback interferometry is a non-destructive technique widely implemented in multiple sensing applications [12]. Light emitted by a laser impinges in a moving target and a part of the scattered light is reinjected inside the laser cavity. This re-injection causes variations in the laser emission power and junction voltage that can be employed to obtain information on the target. Therefore, the laser is used as light source, interferometer and receiver, making OFI sensors generally compact when compared to other sensing devices. Other advantages include its self-alignment, thus avoiding complex alignments required by classical interferometry. In addition, using advantages from the light amplification in the laser cavity where the interferences take place, OFI is sensitive to very low levels of back-scattered optical power. OFI can be considered as a consolidated and mature interferometric technique in mechatronics, typically for velocity, vibration and displacement measurements [13,14] and as an alternative method for multiple biomedical studies [15,16,17]. For a complete overview on the phenomenon and applications related to OFI, we refer the readers to a recent review [18].

OFI’s ability to measure velocity led to its implementation for sensing purposes in diverse fluidic applications. It has been employed in the past for measuring blood flow over skin [19], blood perfusion in tissue [20,21] and drop measurements [22]. Furthermore, this technology allows for reconstruction of velocity profile in channels [9,11,23] depicting good agreement with theory in both cylindrical and rectangular ducts. Therefore, it offers a simple and cost-reduced alternative tool when compared to other methods allowing flow profiling such as optical coherence tomography [24], ultrasonic Doppler flowmetry [25] or laser Doppler anemometry [26]. However, to the best of the authors’ knowledge, the OFI sensing scheme was never implemented for the characterization of multiphase flows.

In OFI applied to flows, light is scattered by moving particles contained in the fluid. Thanks to their properties (i.e., diameter, density), those particles perfectly follow the fluid flow. Thus, the velocity of these tracer particles can be assimilated as the local velocity of the flow. When light scattered by particles in the fluid reenters in the laser, it modulates the spectral properties of the lasing cavity. Consequently, the analysis of the power spectral density provides the fundamental Doppler frequency shift related to velocity. As in classical laser Doppler velocimetry systems, this Doppler frequency is correlated to the fluid’s velocity through a simple relation

(1) $$f_{D}=\frac{2nv\cos\theta }{\lambda }$$

where *n* is the refractive index of the particle’s surrounding medium, *θ* is the angle between the laser propagation axis and the flow velocity vector, *λ* is the laser wavelength and *v* is the fluid’s velocity.

## 3. Experiments

The principle of the experiments is to pump oil and water in a Y-shaped microreactor. Once both immiscible liquids are inside the channel, their interaction produces parallel flows characterized by a continuous interface defining the volume occupied by each fluid. Then, the OFI sensing technique is used to obtain the velocity distribution over a scanned line orthogonal to the flow direction in the channel containing both fluids.

### 3.1. Setup

A custom made Y-shaped microreactor is built in SU8 over a glass substrate using photolithography. The main channel is 11 mm long and the other two channels where the inlets are placed are 7 mm long. The angle between both inlet channels is 60°. All the channels in the microfluidic chip have a 100 µm × 300 µm rectangular cross section (aspect ratio *α* = h/w = 1/3, where *h* represents the channel’s height and *w* is the channel’s width). Oil and demineralized water are injected through the inlets using two independent flow-controlled pumps (Harvard Apparatus Syringe Pump 11 Pico Plus, Holliston, MA, USA). Parallel oil–water flows were obtained with the flow rate of the oil being slower by about one order of magnitude as compared to that of the water. Preliminary measurements performed with an OFI sensor using an infrared laser diode have demonstrated that the effective frequency range of the sensor does not allow measuring the velocity profile in the oil phase pumped at such low flow rates [27]. In addition, in this early work, the optical configuration based on a single focusing lens, induced a relatively large sensing area to the detriment of the resolution. Thus, the sensor’s ability to estimate the localization of the interface was strongly affected. To overcome these limitations, a new OFI flowmeter has been developed.

**Figure 1:**
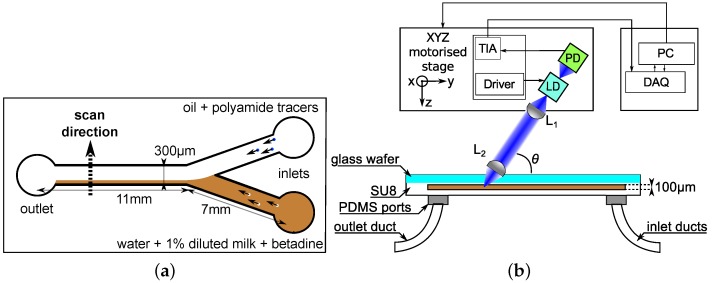
Experimental setup description. (**a**) Physical description of the Y-shaped microreactor (**top** view). The figure describe the location of the two fluids with the respective tracers; and (**b**) optical arrangement and sensor architecture (**lateral** view). The laser (LD) light is focused on the channel using a collimation lens (L1) and a focusing lens (L2) optical arrangement. Oil and water flows are fed through the inlets using two independent syringe pumps

The experimental setup is shown in Figure 1. It consists of a blue-violet laser diode (Panasonic DL-5146-101S, Tokyo, Japan) with a short wavelength *λ* = 405 nm. Kliese et al. demonstrated that OFI flow sensors incorporating lasers with shorter wavelengths are capable of measuring very slow velocities, out of the range that an infrared laser would detect [28]. The laser diode (LD) is coupled to a two-lenses focusing system (both lenses being Thorlabs C240TM-A, Newton, NJ, USA). The lens L1 is used for collimation of the laser beam while the lens L2 is dedicated to the focalization at the microchannel’s center in depth. The laser spot size obtained with this configuration has been calculated using ray tracing softwares and is expected to be around 9 µm in diameter according to the 1/e${}^{2}$ criterion. The variation of the laser power emission induced by the back-scattered and Doppler shifted light (as described in Equation (1)) are collected from the back-facet of the laser using the monitoring photodiode (PD) included in the laser package.

The flows of both fluids in the main channel are visualized using a Digital Microscope Camera (Oowl Tech Ltd., MZ 902, Hong Kong, China). These images are further used to determine the location of the interface by quantifying in terms of pixels the area occupied by oil and water using the upper view of the channel as a reference. It should be noticed that, in the present study, the location of the interface is assumed to be identical along the height of the microchannel.

The piece supporting the laser and lenses is connected to a Labview™-controlled three-axis stage device (Zaber Tech. LSM 50A, Vancouver, BC, Canada) allowing micrometric scanning along the channel’s width of 300 µm.

The signal of the monitoring photodiode is amplified via a custom made transimpedance amplifier (TIA) with a gain of 120 dBV/A. Then, this signal is sampled at 1 MHz and saved into a computer using a National Instruments PCIe-6351 (Austin, TX, USA) data acquisition card (DAQ) and then processed offline using a Matlab (R2013a, The MathWorks, Natick, MA, USA) customized algorithm that is detailed below.

### 3.2. Fluids

Oil (Polydimethylsiloxane, Sigma Aldrich product number 481939, Saint-Louis, MO, USA) and demineralized water are used. Oil’s viscosity and density were determined experimentally to be 28 mPa · s and 0.982 g · cm${}^{-3}$, respectively at 25 °C. Water’s viscosity and density are 1 mPa · s and 1 g · cm${}^{-3}$, respectively. A small concentration (0.4% by mass) of 5 µm tracer polyamide particles (Dantec Dynamics 9080A3011, Skovlunde, Denmark) with density equal to 1.02 g · cm${}^{-3}$ is merged in the oil and 1% by mass of full-cream milk is embedded in the water. The fat particles of milk have proven to be a reliable type of tracers in water flows for the OFI sensing scheme [9], while the polyamide particles, due to their low mass density are not suitable for water flows. During the experiments, a small percentage of betadine (0.2% by mass) was added to water to improve the contrast of both liquids in the images.

### 3.3. Signal Processing

Time domain signals acquired from the internal photodiode are processed so that the power spectral density (PSD) is calculated using Welch’s averaged periodogram method. To enhance the signal-to-noise ratio (SNR) and thus increase the reliability of the Doppler frequency calculation, the spectrum is calculated on the autocorrelation of the OFI signal. The autocorrelation is calculated and normalized so that it is equal to unity at zero lag. We found that the SNR in the PSD of autocorrelated signals is higher by 13 dB as compared to the PSD of raw signals.

**Figure 2:**
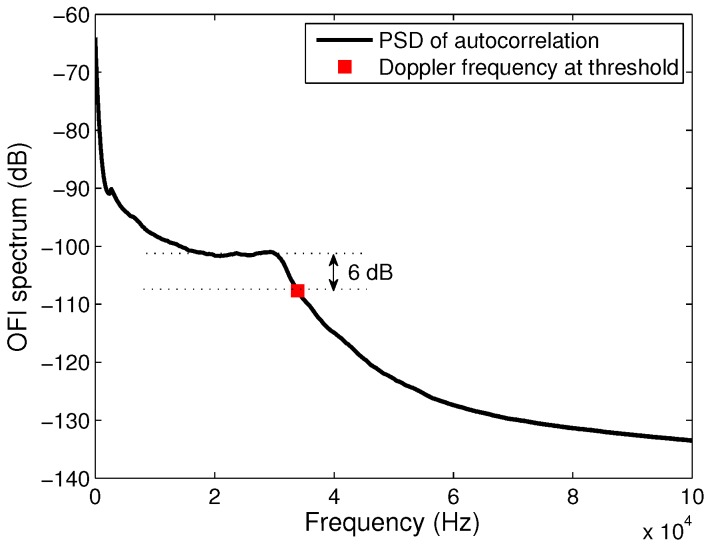
OFI signal spectrum for a solution of diluted milk 98% that was used for the calibration of the sensor. The maximum Doppler frequency calculated using the −6 dB cutoff frequency method is represented by the **red** square.

Since the signal is related to the velocity vector of each particle in the flow, its frequency domain representation shows distribution of power in the low frequency range (Figure 2). The low concentrations of particles in the fluids induces a typical signal’s spectrum with a frequency distribution corresponding to the single scattering regime with a plateau that ends at the maximum Doppler frequency. In the case of single scattering, it is then usually accepted to calculate the maximum velocity from a cutoff frequency determined at a threshold of −3 dB below the plateau of the power spectrum [11]. Because our signal’s spectrum is calculated from the autocorrelation of the signal, then the maximum velocity is found at a cutoff frequency that corresponds to a thresold of −6 dB as depicted in Figure 2, which corresponds to the square of the standard threshold.

## 4. Physical Model

We propose describing the interactions occurring between oil and water when flowing in parallel flow by considering that each fluid can be modelled as a laminar viscous Couette flow. In this case, the Navier–Stokes equation can be reduced to the following expression [29]:

(2) $$\frac{d^{2}v}{dx^{2}}=\frac{1}{\eta }\frac{dP}{dy}$$

where *v* is the axial velocity component of the fluid, $\frac{dP}{dy}$ is the pressure gradient parallel to the walls and to the interface and *η* is the viscosity of the fluid.

**Figure 3:**
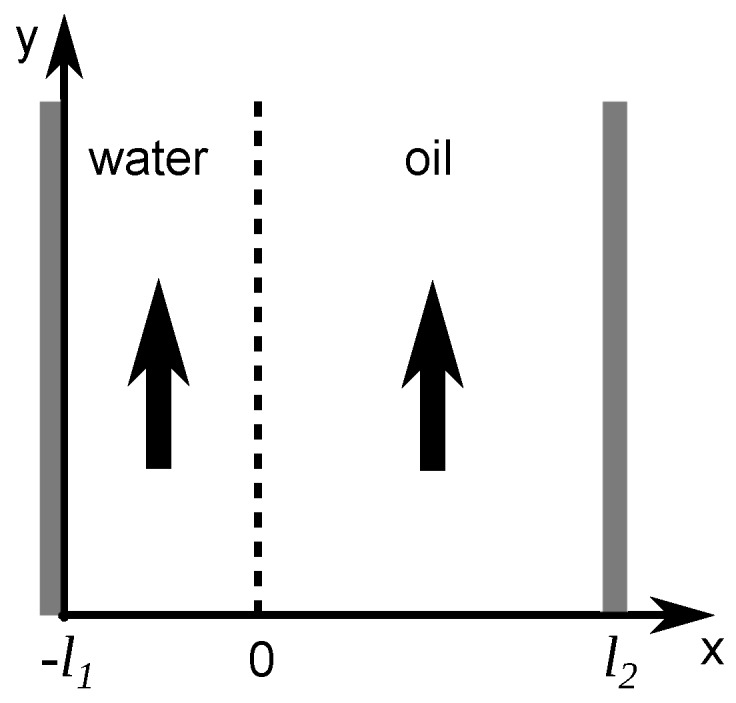
Graphical schematic representation of oil and water in a microchannel. The interface between both liquids is located at position x=0.

Let us consider the scheme of the fluid flows represented in Figure 3, with liquid 1 as water and liquid 2 as oil. The microchannel of width $w=l_{2}+l_{1}$ contains both immiscible fluids and the interface between them is located at a transverse position x=0 along the channel width. Considering a constant pressure gradient, solving Equation (2) for each phase leads to the following formulation for water and oil, respectively:

(3) $$\frac{d^{2}v_{1}}{dx^{2}}=\frac{1}{\eta_{1}}\frac{dP}{dy}=A_{1}$$

(4) $$\frac{d^{2}v_{2}}{dx^{2}}=\frac{1}{\eta_{2}}\frac{dP}{dy}=A_{2}$$

Equations (3) and (4) lead to the following solutions for each phase:

(5) $$v_{1}\left(x\right)=\frac{A_{1}x^{2}}{2}+B_{1}x+C_{1}$$

and

(6) $$v_{2}\left(x\right)=\frac{A_{2}x^{2}}{2}+B_{2}x+C_{2}$$

where $B_{i}$ and $C_{i}$ are constants that are extracted by taking into consideration that both fluids comply with the no-slip condition in the walls. Thus, null velocities at the walls serve as boundary conditions leading to the solutions of Equations (5) and (6). Considering the water–wall location on the *x*-axis as $-l_{1}$ and the oil-wall location as $l_{2}=w-l_{1}$, the boundary conditions are: $v_{1}\left(-l_{1}\right)=v_{2}\left(l_{2}\right)=0$. The velocity distribution in the microchannel is then given by

(7) $$v_{1}\left(x\right)=\frac{A_{1}x^{2}}{2}+\left[\frac{v_{i1}}{l_{1}}+\frac{A_{1}}{2}l_{1}\right]x+v_{i1}\;\mathrm{for}\;-l_{1}<x<0$$

and

(8) $$v_{2}\left(x\right)=\frac{A_{2}x^{2}}{2}-\left[\frac{v_{i2}}{l_{2}}+\frac{A_{2}}{2}l_{2}\right]x+v_{i2}\;\mathrm{for}\;0<x<l_{2}$$

where $v_{1}$ and $v_{2}$ are the axial velocities of water and oil at a given transverse location *x*, respectively, and $v_{i1}$ and $v_{i2}$ are the axial velocity components of water and oil at each side of the interface.

## 5. Results and Discussion

As a first step, a characterization of the measurement system is performed. Diluted full-cream milk (2% by mass) is pumped inside the microreactor at 10 µL · min${}^{-1}$ through both inlets and the laser position and orientation are set in order to obtain the best signal-to-noise ratio. In this optimal configuration, the angle between the optical axis and the flow velocity is *θ* = 70°. A typical power spectral distribution of a the signal acquired at the center of the channel is shown in Figure 2. The SNR is around 45 dB and the Doppler frequency correlated to the maximum velocity of milk in the channel flowing at 20 µL · min${}^{-1}$ (10 µL · min${}^{-1}$ provided through each inlet) is 6 dB below the plateau.

We propose investigating experimentally the oil-water parallel flows under two different situations.

**Figure 4:**
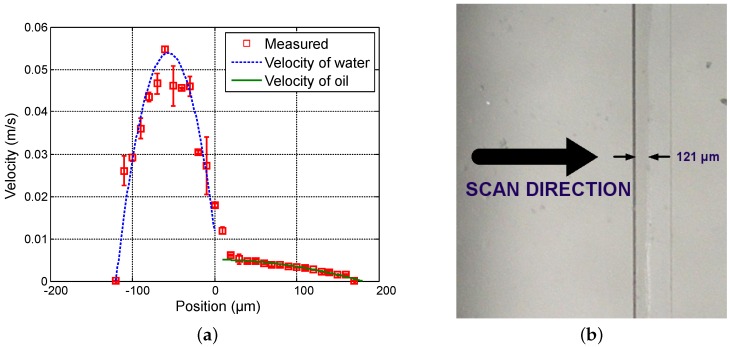
Experimental results for $Q_{\mathrm{water}}$ = 20 µL · min${}^{-1}$ and $Q_{\mathrm{oil}}$ = 3 µL · min${}^{-1}$. (**a**) Measured profile (squares) and theoretical profile for water (dashed line) and for oil (solid line); and (**b**) reference image of the main channel with indication of the interface location.

**Figure 5:**
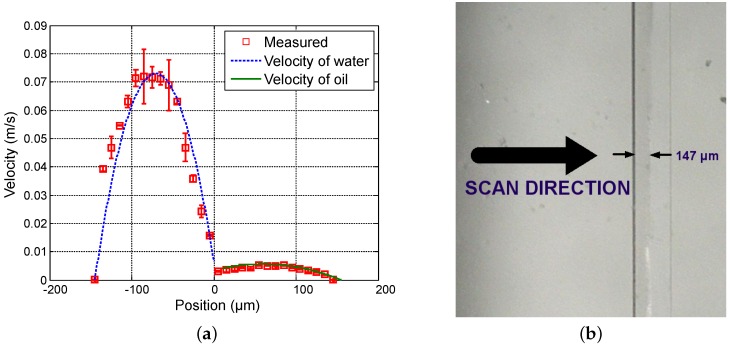
Experimental results for $Q_{\mathrm{water}}$ = 35 µL · min${}^{-1}$ and $Q_{\mathrm{oil}}$ = 3 µL · min${}^{-1}$. (**a**) Measured profile (squares) and theoretical profile for water (dashed line) and for oil (solid line); and (**b**) reference image of the main channel with indication of the interface location.

**Figure 6:**
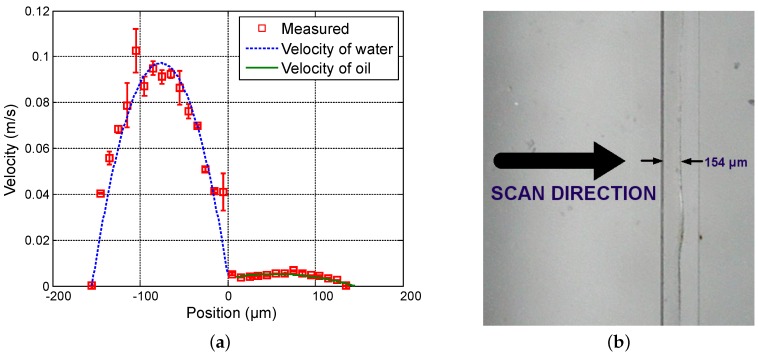
Experimental results for $Q_{\mathrm{water}}$ = 50 µL · min${}^{-1}$ and $Q_{\mathrm{oil}}$ = 3 µL · min${}^{-1}$. (**a**) measured profile (squares) and theoretical profile for water (dashed line) and for oil (solid line); and (**b**) reference image of the main channel with indication of the interface location.

First, in order to vary the position of the interface between both fluids, the flow rate of water ($Q_{\mathrm{water}}$) varies from 20 µL · min${}^{-1}$ to 50 µL · min${}^{-1}$ in steps of 15 µL · min${}^{-1}$ while the flow rate of oil ($Q_{\mathrm{oil}}$) is fixed at 3 µL · min${}^{-1}$. Figure 4, Figure 5 and Figure 6 show the measured velocity profiles associated with the oil-water parallel flows in the microchannel. The square measurement points represent the averaging of the maximum velocity values measured over eight consecutive scans and the error bars represent the standard deviation of the maximum velocities at the same position. The locations of the interface, determined by image analysis, are reported for these profiles. The velocity profiles show that each fluid develops its own profile as stated by Pohar et al. [30]. In addition, at Figure 4, one can see that a slipping phenomenon exists at the interface. The velocity is not null due to the dragging effect of water on oil, while, for higher flow rate ratios (Figure 5 and Figure 6), the dragging effect is much less notable. In the case represented in Figure 4, the water flow affects the oil flow in a way that the oil reaches its maximum velocity in the vicinity of the interface. This behavior is typical of Couette flows, in which it is considered that each liquid flows in between two plates, one of which is moving—in this case, the fluids interface. For the configurations represented in Figure 5 and Figure 6, our measurements indicate a small slipping at the interface, as velocity values have a local minimum there.

**Figure 7:**
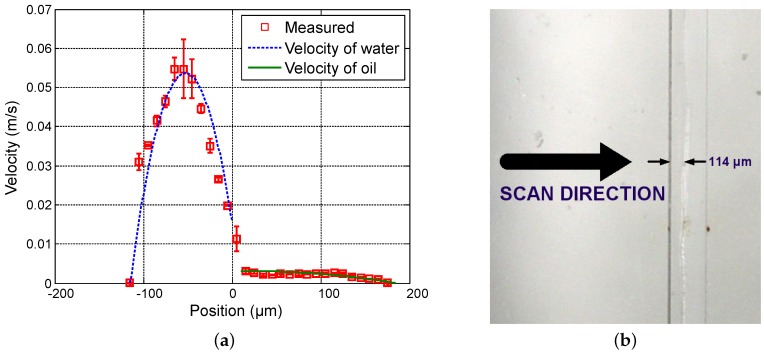
Experimental results for $Q_{\mathrm{water}}$ = 20 µL · min${}^{-1}$ and $Q_{\mathrm{oil}}$ = 1.5 µL · min${}^{-1}$. (**a**) Measured profile (squares) and theoretical profile for water (dashed line) and for oil (solid line); and (**b**) reference image of the main channel with indication of the interface location.

**Figure 8:**
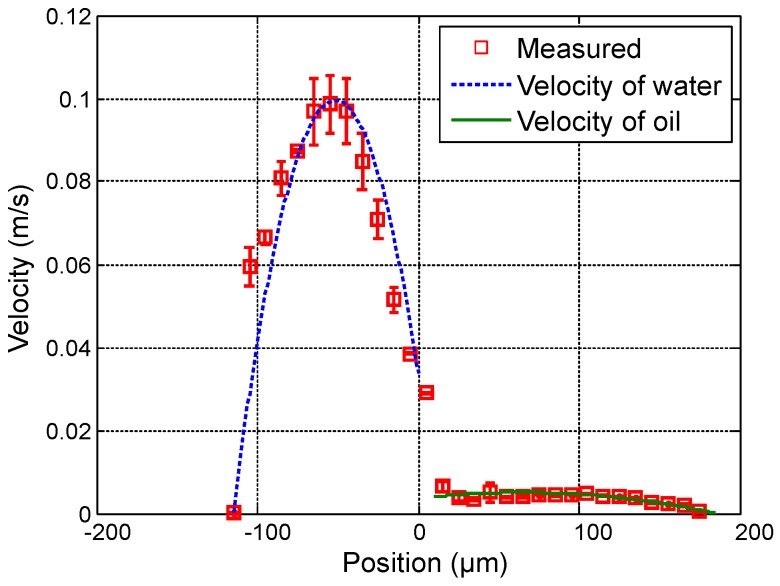
Measured profile with $Q_{\mathrm{water}}$ = 40 µL · min${}^{-1}$ and $Q_{\mathrm{oil}}$ = 3 µL · min${}^{-1}$. The reference image is the same as Figure 7b.

**Figure 9:**
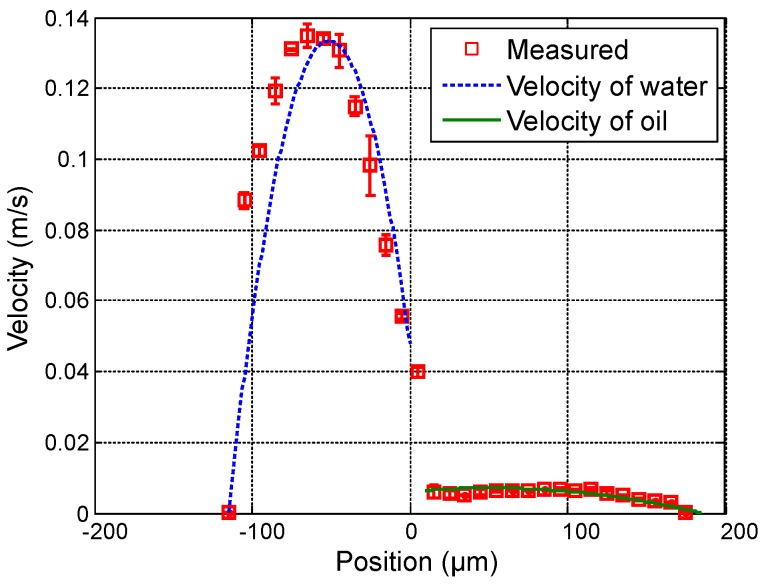
Measured profile with $Q_{\mathrm{water}}$ = 60 µL · min${}^{-1}$ and $Q_{\mathrm{oil}}$ = 4.5 µL · min${}^{-1}$. The reference image is the same as Figure 7b.

Furthermore, scan measurements are carried out aiming at profiling velocity fields of oil-water parallel flows for which the ratio of flow rates is kept constant. Measurements are performed with $Q_{\mathrm{oil}}$ varying from 1.5 µL · min${}^{-1}$ to 4.5 µL · min${}^{-1}$ in steps of 1.5 µL · min${}^{-1}$, and, proportionally, $Q_{\mathrm{water}}$ is varying as follows: 20, 40 and 60 µL · min${}^{-1}$. Figure 7, Figure 8 and Figure 9 show measured velocity profiles for oil and water when the ratio of flow rates remains constant. Square points and errorbars are calculated from eight scans. Measurements enable the verification that the interface location remains at the same location as the flow rates are varied proportionally, and thus the fraction of volume occupied by each fluid in the microchannel remains constant. These findings confirm the relevance of the OFI technique when implementing in two-phase parallel flows.

The parameters of the model used to fit the model curves to the experimentally obtained values plotted in Figure 4, Figure 5, Figure 6, Figure 7, Figure 8 and Figure 9 are shown in Table 1. As depicted in the figures, a fairly good agreement is found between theoretical profiles plotted after Equations (7) and (8), and experimentally measured profiles. Thus, the theoretical approximation considering two independent viscous fluids interacting in the microchannel is suitable to describe the system’s hydrodynamics, even when this interaction makes the fluid behave as Couette flows. The negative values in parameters $A_{1}$ and $A_{2}$ denote the presence of profiles in which the pressure gradient is favorable.

In order to definitively validate the technique, an integration of the velocity distribution can serve to obtain the flow rates imposed in the inlets for every tested configuration. Shah and London [31] proposed an expression in the case of a rectangular microchannel to obtain the volumetric flow rate from the velocity distribution. Considering that the measured profile is scanned from one wall until the other and that our laser detects a maximum frequency in the center in depth of the channel, the flow rates are calculated from the mean values represented in square points in the graphs using the following approximation for a channel with aspect ratio *α* = 1/3:

(9) $$Q=\int_{-h/2}^{h/2}v_{\mathrm{meas}}\left(x\right)\left[1-{\left(\frac{z}{h}\right)}^{2}\right]dz$$

Calculations obtained from the integration in Equation (9) are represented in Table 2. Again, a fairly good agreement is found when compared to the flow rates imposed for each configuration with errors of around 7.5% in the worst case of very high flow rates and of only a few percent in other situations.

Results obtained with the optical feedback interferometry technique are in good agreement with the Couette flow theoretical model developed and with the real parameters imposed at the inlets of the microreactor. This experimental demonstration may provide a new tool to assess in the future the velocity profiles in the case of droplets flowing in a mcirochannel.

## 6. Conclusions

Optical feedback interferometry has been proposed and tested for profiling velocity of oil–water parallel flows in a microchannel. It is shown how the knowledge of the velocity profiles is essential to analyze the possible effects of one liquid phase in the other. The Couette flow approximation has been found to be a good representation of the liquid-liquid two-phase system’s hydrodynamics. The measured profiles are in good agreement with theoretical profiles conceived to represent two viscous immiscible fluids. The integration of the velocity distribution confirms the accuracy of our measurements. This work is remarkable progress for the deployment of optical feedback interferometers in multiphasic microscale systems or for the observation of complex phase interactions such as droplets, fingers or dispersed flows. 

## Figures and Tables

**Table 1:** Fitting parameters used in Equations (7) and (8).

$Q_{\mathrm{water}}$	$Q_{\mathrm{oil}}$	$A_{1}$	$A_{2}$	$v_{i1}$	$v_{i2}$
(µL · min${}^{-1}$)	(µL · min${}^{-1}$)	(m${}^{-1}$ · s${}^{-1}$)	(m${}^{-1}$ · s${}^{-1}$)	(m · s${}^{-1}$)	(m · s${}^{-1}$)
20	3	−2.65 × 10${}^{-7}$	−2.95 × 10${}^{-5}$	11.9 × 10${}^{-3}$	5.1 × 10${}^{-3}$
35	3	−2.65 × 10${}^{-7}$	−1.25 × 10${}^{-6}$	6.5 × 10${}^{-3}$	3.4 × 10${}^{-3}$
50	3	−3.15 × 10${}^{-7}$	−1.25 × 10${}^{-6}$	5.12 × 10${}^{-3}$	3.4 × 10${}^{-3}$
20	1.5	−2.75 × 10${}^{-7}$	−2.35 × 10${}^{-6}$	15.7 × 10${}^{-3}$	3.0 × 10${}^{-3}$
40	3	−4.95 × 10${}^{-7}$	−6.95 × 10${}^{-6}$	33.7 × 10${}^{-3}$	3.6 × 10${}^{-3}$
60	4.5	−6.60 × 10${}^{-7}$	−1.25 × 10${}^{-6}$	47.1 × 10${}^{-3}$	3.4 × 10${}^{-3}$

**Table 2:** Flow rates calculated by integrating the experimental velocity profile Equation (8).

Actual Total Flow Rate ($Q_{\mathrm{water}}$ + $Q_{\mathrm{oil}}$)	Measured Total Flow Rate	Relative Error
(µL · min${}^{-1}$)	(µL · min${}^{-1}$)	(%)
21.5 (20 + 1.5)	22.3	4.02
23 (20 + 3)	23.4	1.79
38 (35 + 3)	36.4	4.08
43 (40 + 3)	42.1	1.86
53 (50 + 3)	51.8	2.19
64.5 (60 + 4.5)	59.9	7.5
